# Advances in Poultry RNA-Omics Research: Technologies, RNA Information Layers, and Applications in Complex Traits

**DOI:** 10.3390/ani16172700

**Published:** 2026-08-31

**Authors:** Wenbin Dao, Simeng Zhang, Tao Zhang, Xinyang Fan, Yongwang Miao

**Affiliations:** Institute of Animal Genetics and Breeding, College of Animal Science and Technology, Yunnan Agricultural University, Kunming 650201, China; dwbin666@126.com (W.D.); 18398513250@163.com (S.Z.); TTyyrlyk@163.com (T.Z.)

**Keywords:** poultry, RNA-omics, long-read RNA sequencing, epitranscriptomics, single-cell and spatial transcriptomics, complex traits

## Abstract

RNA-omics technologies have expanded poultry transcriptome research beyond tissue-level gene abundance. They now resolve intact transcripts, RNA chemical modifications, cellular origins, and spatial locations. This review centers on a core question: what specific, previously inaccessible RNA information does each RNA-omics technology provide? Based on their primary measurement targets, we categorize these technologies into four groups: bulk transcriptome and small RNA sequencing for analyzing gene expression and regulatory RNA networks; long-read and direct RNA sequencing for resolving transcript isoforms; epitranscriptomic methods for detecting RNA modifications; and single-cell and spatial transcriptomics for identifying the cellular origins and spatial distributions of signals. Methods for resolving RNA structure, RNA–protein interactions, and translation status (e.g., structure probing, CLIP-type mapping, and ribosome profiling) remain in their infancy in poultry, leaving translation and RNA–protein regulation largely unmeasured in tissues such as the oviduct, the lipogenic liver and nucleated erythrocytes. For each category, we discuss its direct measurement targets and primary outputs, the additional information it provides compared to established methods, representative applications in poultry, and its inherent limitations. Building on this framework, we discuss how to select and combine technologies based on specific research questions and summarize their applications in studies of production performance and product quality, reproduction, and health and resilience. Different technologies measure distinct types of RNA features and are not simply interchangeable. Future research must place greater emphasis on matching technologies to scientific questions, integrating complementary data, and improving three foundations: the annotation of poultry transcripts and non-coding RNAs, standardized analytical pipelines, and functional validation systems.

## Simple Summary

Most poultry studies measure average amounts of ribonucleic acid in a tissue. This approach can identify genes that become more or less active, but it cannot fully explain which form of a gene is produced, which cells produce it, where those cells are located, or whether chemical marks affect how the molecule behaves. This review compares the main methods used to study these features and examines how they have been applied to poultry production and product quality, reproduction, and responses to disease and environmental challenges. Each method reveals a different part of ribonucleic acid biology, so the newest method is not always the most suitable one. Combining methods can provide stronger evidence, particularly when measurements come from the same animals and address the same question. Even so, many reported links between molecular changes and poultry traits remain predictions or correlations rather than directly tested relationships. On their own, such correlations cannot be used to choose breeding animals, because they do not show how much of the variation is inherited or whether the molecule causes the trait. Better reference resources, carefully matched samples, consistent analysis, and experiments that test specific findings are still needed. These improvements should make poultry research more reliable and provide a stronger basis for breeding, health management, and efficient production.

## 1. Introduction

Since the application of high-throughput RNA sequencing, transcriptome analysis has become the primary approach for investigating the molecular basis of important traits in poultry. Bulk RNA sequencing and conventional differential expression analyses are widely used in studies of muscle growth, fat deposition, reproduction, immune responses, and environmental stress [1,2,3,4]. By measuring relative RNA abundance in tissues, these methods have established a research paradigm centered on gene expression changes. They have also provided substantial evidence for understanding the regulatory basis of economically important poultry traits. While these methods remain indispensable today, their results primarily reflect the average levels of diverse cells and their RNA signals within a tissue.

Relying solely on RNA abundance is insufficient to identify all RNA features relevant to phenotype formation [5,6]. A change in the expression level of a given gene might stem from transcriptional regulation within a relatively stable cell population, or it could be driven by shifts in cellular composition [7], altered transcript usage [8], or differences in RNA stability [9]. Conventional analyses generally cannot reliably determine which full-length isoforms a gene produces, whether an RNA carries chemical modifications, which specific cell types emit the expression signal, or where those cells are located within the tissue. They also cannot directly ascertain which proteins bind to the RNA or whether the RNA is actively being translated. These are not merely supplementary details to gene expression levels, but fundamentally distinct RNA features [6,10]. Without differentiation, variations with entirely different biological implications may be grouped into a single category of expression outcomes. These sources also differ in how far they can travel toward genetic improvement. Tissue-level RNA abundance responds strongly to diet, age, stress and housing, so an expression contrast measured in one management setting mixes heritable and environmental components, and only the heritable component behaves as a selectable molecular phenotype: in cattle the genetic component of expression and splicing explains a large share of complex-trait heritability [11], whereas expression heritability itself varies widely among genes and tissues in pigs [12]. Until these components are partitioned by eQTL or sQTL mapping or by sampling related individuals, an RNA contrast cannot identify a stable genetic signal or predict the response to selection.

Recently developed RNA-omics technologies have introduced many previously difficult-to-measure RNA features into poultry research. Long-read sequencing can resolve complete or near-complete transcript structures, while direct RNA sequencing can preserve some native molecular signals of the original RNA [13,14]. Epitranscriptomic methods are used to detect RNA chemical modifications [15,16], and single-cell, single-nucleus, and spatial transcriptomics can further determine the cellular origins and spatial distributions of expression signals within tissues [17,18]. Methods analyzing RNA structure, RNA–protein interactions, and translation states extend research beyond RNA abundance into functional domains [19,20]. These advances are particularly important for poultry research. Compared to humans and mice, the annotation of poultry transcripts and non-coding RNAs remains incomplete, and GC-rich sequences alongside microchromosomal regions have long complicated genome assembly and transcript annotation [21]. This gap has a direct breeding consequence: avian microchromosomes are gene-dense and carry regulatory elements and trait-associated variants, so transcripts and regulatory RNAs that are missing or misplaced in the annotation are also missing from the candidate functional variants available to genomic prediction [21,22]. Because many RNA-omics methods were initially established in mammals, their applicability, analytical parameters, and result interpretation in poultry require species-specific validation. The assumptions that fail are concrete: marker panels transferred from mouse or human often do not mark the homologous avian cell type; birds have no lymph nodes, diversify the B-cell repertoire in the bursa of Fabricius, and use nucleated erythrocytes and thrombocytes; de novo lipogenesis is concentrated in the liver rather than in adipose tissue; and reproduction operates through a single functional ovary, a follicular hierarchy and an egg-assembling oviduct [23,24]. Following the recent establishment of long-read transcript catalogs and multi-tissue expression resources in chickens [25,26,27], it is now necessary to systematically evaluate what information different RNA-omics technologies can provide in poultry research, what their limitations are, and how these methods should be selected and combined.

Existing poultry reviews largely organize their content by trait domain or broad omics categories [1,3,4], but rarely systematically compare the direct measurement signals, added information, and inferential limitations of different RNA technologies [28,29]. This can inadvertently portray these technologies as a linear progression of increasing complexity, obscuring the fact that they answer fundamentally different biological questions. This review is therefore structured around the core theme of RNA information layers. Here “layers” refers to different types of measurable RNA features rather than a technological hierarchy: tissue-averaged abundance, regulatory RNA profiles, full-length transcript isoforms, chemical modifications, cellular origins, spatial locations, RNA structures, RNA–protein interactions, and translation states. Based on this framework, we first outline developments in bulk RNA and regulatory RNA analyses, long-read sequencing, epitranscriptomics, and single-cell and spatial transcriptomics, and also cover emerging methods that have seen limited application in poultry. We then compare technology selection and combination strategies according to specific research questions, and review their applications in studies of production performance and product quality, reproduction, and health and resilience. Chickens serve as the primary reference species due to the abundance of available evidence [21,30,31]. Studies in ducks, geese, and turkeys are included when they contribute complementary technological or biological insights. Finally, we summarize common limitations in annotation resources, experimental design, computational analysis, and functional validation, and propose priority directions for future development. Throughout this review, we carefully distinguish among direct measurement readouts, computational inferences, and experimentally validated mechanisms. Where a conclusion rests on computational inference alone, for example a network built from sequence complementarity and expression correlation, a pseudotime ordering, or a ligand–receptor pairing taken from a mammalian database, we label it a candidate relationship and state what would test it; only studies applying a perturbation, such as writer or reader manipulation, isoform-specific interference, or a validated organoid or in vivo assay, are described here as functional evidence.

## 2. Review Methodology

This narrative review searched PubMed, the Web of Science Core Collection, and Google Scholar for English-language studies published from database inception to 1 July 2026. Search terms were constructed by combining poultry-related keywords (chicken OR *Gallus gallus* OR duck OR goose OR turkey OR quail OR broiler OR hen OR avian OR poultry) with terms corresponding to major RNA information layers and related technologies. These included non-coding RNA and regulatory RNA, alternative splicing and transcript isoforms, long-read and direct RNA sequencing, RNA modification and epitranscriptomics, single-cell and spatial transcriptomics, RNA structure, RNA–protein interaction, and ribosome profiling. When searching for applied studies, these terms were further combined with keywords related to production and product quality, reproduction, and health and resilience. Additional studies were identified by screening the reference lists of relevant papers and reviews. The layer-specific term sets, the PubMed counts they return and the number of records cited from each block are reported in Table 1.

Records were included when they were in English with a full text available; reported original data, a benchmarking analysis, a resource or a review; addressed at least one of the RNA information layers defined above; and were either performed in a poultry species or, for methodological work in mammals or other systems, cited only to establish a technical principle, a limitation or a validation requirement. Records were excluded when RNA measurement was only an assay readout, when a non-poultry study made no methodological point, or when a preprint had been superseded by its peer-reviewed version. Poultry methodological and resource studies were used to define the measurement targets of each technology and what specific RNA information they render observable. Applied poultry studies were included when they successfully linked clear RNA features to poultry biology or one of the three aforementioned trait domains. Fundamental methodology studies and analytical benchmarking studies conducted in mammals or other model systems were included only to illustrate technological principles, limitations, or validation requirements; these studies are not presented as direct evidence in poultry. Comparative terms such as “most,” “few,” and “rare” refer to the counts in Table 1, not to impressions. Those counts make the imbalance explicit: within the poultry term set, regulatory and non-coding RNA returns 1411 records and long-read or direct RNA sequencing 430, whereas single-cell and single-nucleus sequencing returns 624, m^6^A profiling 91, spatial transcriptomics 15, ribosome profiling 13 and CLIP-type RNA–protein mapping 2. They are lower bounds set by the term sets rather than bibliometric censuses, and identified records were not screened through a formal systematic-review cascade, because this is a narrative technology-oriented review rather than a systematic review of one answerable question.

## 3. RNA-Omics Technologies and the RNA Information Layers They Reveal

**Figure 1:**
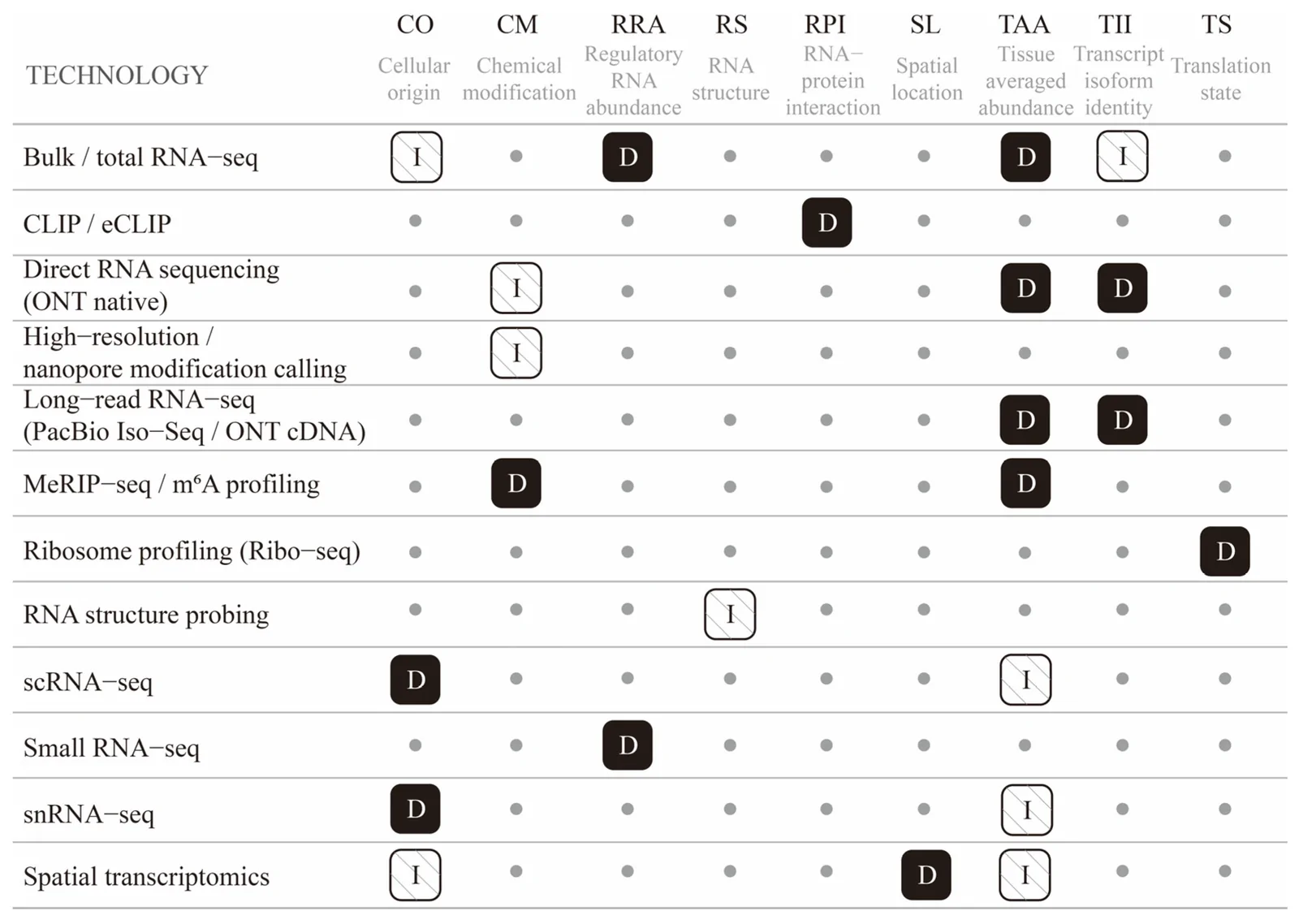
Coverage of RNA information layers by poultry RNA-omics technologies, and how each layer is obtained. Rows list technologies and columns list layers of RNA information; the ordering of either axis does not imply a sequence, hierarchy, or level of sophistication. Each cell states whether the technology measures that layer directly (filled, D), obtains it only by inference or aggregation from its own data (hatched, I), or does not provide it (dot). Any cell is therefore a valid entry point: a technology is appropriate when its direct readout answers the question being asked. A single layer can be reached by technologies of very different resolution, and the direction of that difference reverses between layers. Single-cell methods infer tissue-averaged abundance by aggregation, while bulk sequencing measures that layer directly and infers cellular composition by deconvolution. The corresponding research questions, together with the current implementation status of each technology in poultry, are given in Table 6.

The technologies discussed here provide complementary RNA information rather than forming a unidirectional, simple-to-complex technological pathway (Figure 1). The order of presentation below follows how widely each technology is used in poultry, not how advanced it is, and it is not a recommended sequence: which technology is appropriate is set by the biological question and the RNA feature it requires, so a well-replicated bulk experiment can be the correct design where a single-cell experiment would answer a different question expensively. Short-read RNA sequencing quantifies gene- and exon-level abundance and can identify unannotated exons and splice junctions, but fragmented reads generally struggle to reliably reconstruct full-length transcripts, and tissue-level signals are inevitably influenced by cellular composition [2,32]. The consequences are specific: splice junctions are recovered but not their combinations, so genes with several alternative exons collapse into ambiguous isoform assignments; long 3′ untranslated regions and alternative polyadenylation sites are truncated as coverage decays towards transcript ends; and GC-rich exons, over-represented on avian microchromosomes, are under-covered during amplification, so exon-level quantification is least reliable where poultry annotation is weakest [21,33]. Small RNA sequencing and total transcriptome sequencing expand the analytical scope to miRNAs, lncRNAs, and circRNAs, thereby capturing regulatory RNA information that conventional gene expression analyses often miss. Long-read sequencing can further resolve intact or nearly intact transcript isoforms, alternative splicing, and 3′-end usage [13,14]. Antibody enrichment-based methods and native RNA analyses, such as direct RNA sequencing, can be used to profile RNA modifications like m^6^A [34]. Single-cell and spatial transcriptomics further pinpoint the cellular origins of expression signals and their spatial distributions within tissues [17]. Information regarding RNA structure, RNA–protein interactions, and translation states currently remains at the frontier of poultry RNA-omics research, with limited applications to date. The following sections introduce these technologies sequentially based on the aforementioned information types, beginning with the most widely applied methods in poultry. For each technology category, we outline its direct measurement signals, the additional information it provides over existing methods, representative applications in poultry, and main limitations. The measurement and practical profiles of sequencing and modification technologies are summarized in Table 2 and Table 3, whereas those of cellular, spatial, interaction, translation, and RNA-structure technologies are summarized in Table 4 and Table 5. The text refers repeatedly to two reference resources: the multi-tissue long-read transcript annotation in chickens [26] and the catalog of associations among cis-regulatory variants, gene expression, splicing, and 3′-UTR usage established by the ChickenGTEx project [25,27].

**Table 2:** Measurement profile of poultry RNA-omics sequencing and modification profiling technologies.

Technology	RNA Input	Primary Signal	Main Analytical Output	Additional Information Revealed	Representative Poultry Applications
Bulk/total RNA-seq	Total or poly-A RNA (tissue)	Short reads	Gene/transcript abundance; DE; co-expression	Genome-wide expression and regulatory-network membership	Myogenic and fat networks [35,36,37,38,39,40]; reviews [1,3,4]
Small RNA-seq	Size-selected small RNA	Short reads	Mature miRNA abundance	miRNA layer of regulatory networks	miRNA surveys in health/production [41]; embryonic muscle m^6^A–miRNA [42]
Long-read RNA-seq (PacBio Iso-Seq/ONT cDNA)	Full-length cDNA	Long reads	Isoform catalogue; splicing/TSS/APA; transcript-level abundance	Complete or near-complete transcript identity	19-tissue chicken isoforms [26]; embryonic heart [43]; benchmark [44]
Direct RNA sequencing (ONT native)	Native RNA	Ionic current on nanopore	Isoform + candidate modification from same read; transcript-level abundance	Native-molecule signal; joint isoform/modification	Caecal m^6^A/m^5^C after *C. jejuni* [45]; method [46]
MeRIP-seq/m^6^A profiling	Fragmented RNA + m^6^A antibody	Enriched methylated reads	m^6^A-enriched regions	Transcriptome-wide modification state	Laying [47]; follicle [48]; tumour liver [49]
High-resolution/nanopore modification calling	Native or chemically treated RNA	Basecalling deviation/chemistry	Candidate single-base sites	Higher-resolution modification localisation	β-actin zipcode fine-mapping [50]

**Table 3:** Practical profile of poultry RNA-omics sequencing and modification profiling technologies. Input and cost entries are indicative order-of-magnitude guides from the sources cited in Table 2 and in the text. They are not vendor quotations, vary with provider, country, protocol and depth, and should be re-checked locally.

Technology	Typical Input and Sample Requirement	Relative Cost and Throughput	Inter-Laboratory Reproducibility	Strengths	Limitations
Bulk/total RNA-seq	0.1–1 µg total RNA, RIN ≥ 7; tolerant of frozen tissue	Lowest tier; baseline for comparison; high multiplexing	High; limited mainly by annotation version and pipeline choice	Deep, reproducible, low cost, comparable	Tissue average; gene-level; networks inferred
Small RNA-seq	0.1–1 µg total RNA with small RNA fraction preserved	Low tier; baseline plus a dedicated protocol step	Moderate; adapter-ligation bias differs between kits	Direct miRNA quantification	Requires dedicated protocol; target prediction inferred
Long-read RNA-seq (PacBio Iso-Seq/ONT cDNA)	50–500 ng intact poly(A) RNA or full-length cDNA; degradation is the main failure mode	Intermediate tier, several-fold baseline per sample; lower depth	Moderate; isoform catalogues shift with pipeline and filtering [13,44]	Resolves isoforms short reads miss	Not all reads full-length; tool-dependent; lower depth
Direct RNA sequencing (ONT native)	Intact native poly(A) RNA, typically ≥ 500 ng per flow cell	Intermediate to high tier; low throughput per flow cell	Low to moderate; modification calls depend on model version [51]	No cDNA/PCR bias; long native reads	Model-dependent modification calls; error; 3′ bias
MeRIP-seq/m^6^A profiling	Tens to hundreds of µg total RNA per immunoprecipitation, plus a matched input library	Intermediate tier, effectively doubled by the paired input library	Low; antibody lot, immunoprecipitation conditions and peak-calling parameters give only partial overlap between studies [34]	Established, transcriptome-wide	Region-level (not single-base); semi-quantitative; antibody-dependent
High-resolution/nanopore modification calling	As for direct RNA sequencing, or chemically treated intact RNA	Intermediate to high tier; requires reference standards	Not established in birds; tools disagree on candidate sites [45,51,52]	Approaches single-base resolution	Early in birds; tools diverge [45]

**Table 4:** Measurement profile of poultry cellular, spatial and interaction analysis technologies.

Technology	RNA Input	Primary Signal	Main Analytical Output	Additional Information Revealed	Representative Poultry Applications
scRNA-seq	Dissociated single cells	Per-cell short reads (3′/5′)	Cell types and states	Cellular origin of a tissue signal	Heart/limb/retina [53,54,55]; immune maps [56,57,58]
snRNA-seq	Isolated nuclei	Per-nucleus reads	Cell types and states (incl. hard-to-dissociate)	Cell origin in fibrous tissue	Muscle satellite-cell subset (RUNX1) [59]
Spatial transcriptomics	Tissue section	Spot/region-resolved reads	Spatially resolved expression	Tissue position of a signal	Developing chicken heart [54]; wooden-breast [60]; grower breast muscle [56]
CLIP/eCLIP (RBP mapping)	RNA–protein complexes	Crosslinked RBP-bound fragments	RNA–protein binding sites	Which protein binds an RNA	Chicken embryonic heart CELF1 [61]; mammalian method reference [62]
Ribosome profiling (Ribo-seq)	Ribosome-protected fragments	Footprint reads	Ribosome occupancy; translation efficiency	Whether RNA is translated	Mammalian method reference [19]
RNA structure probing	Chemically/enzymatically treated RNA	Reactivity signal	Structure model	RNA secondary/tertiary state	No poultry transcriptome-wide application identified

**Table 5:** Practical profile of poultry cellular, spatial and interaction analysis technologies. Input and cost entries follow the convention stated for Table 3.

Technology	Typical Input and Sample Requirement	Relative Cost and Throughput	Inter-Laboratory Reproducibility	Strengths	Limitations
scRNA-seq	Fresh tissue dissociated to a viable single-cell suspension, commonly ≥ 70–80% viability, thousands of cells per sample	Highest tier, commonly about an order of magnitude above a bulk library per sample	Moderate; dissociation protocol and ambient RNA dominate batch differences [63]	Resolves heterogeneity	3′/5′ gene-level; no isoform/modification; mammalian markers
snRNA-seq	Fresh or frozen tissue; nuclei isolated without a viability requirement	Highest tier, comparable to scRNA-seq	Moderate; nuclei isolation buffer changes the recovered composition [64]	Works where dissociation fails	Nuclear RNA only; isolation bias
Spatial transcriptomics	Intact tissue section of defined area with RNA quality preserved through fixation and cryosectioning	Highest tier per section, plus imaging and instrument access	Low to moderate; capture efficiency varies between sections and platform generations [65,66]	Retains anatomy	Spot ≠ single cell; few avian tissues
CLIP/eCLIP (RBP mapping)	Millions of crosslinked cells plus an antibody validated for immunoprecipitation, or a tagged RBP system	Intermediate in reagents, high in development effort	Not established in birds; antibody availability is the limiting factor [62]	Direct interaction map	One chicken application (CELF1, embryonic heart); no transcriptome-wide avian binding map
Ribosome profiling (Ribo-seq)	Fresh tissue or cells with translation arrested within seconds; species-specific rRNA depletion	Intermediate to high tier; two libraries per sample (footprint and matched RNA)	Moderate in mammals; not benchmarked in poultry [19]	Translational-state readout	Sparse avian evidence
RNA structure probing	µg amounts of intact RNA per condition and per chemistry, with a matched untreated control	Intermediate tier; requires probing chemistry expertise	Not established in birds; no avian benchmark dataset [20,67]	Structure–function link	Not established transcriptome-wide in birds

**Table 6:** Correspondence between research questions, RNA-omics technologies, and their implementation status in poultry.

Research Question	Main Technology	RNA Information Obtained	Poultry Implementation Status
Which genes or transcripts change in abundance?	Bulk or total RNA-seq	Tissue-averaged gene or transcript abundance	Established: widely applied across muscle, adipose, reproductive and immune tissues
Which regulatory RNAs change?	Small-RNA or total-RNA sequencing	miRNA, lncRNA or circRNA abundance and inferred networks	Established: widely applied, especially in muscle, fat and infection studies
Which isoforms or transcript boundaries change?	Long-read RNA sequencing	Full-length or near-full-length isoforms, splice forms and transcript ends	Emerging: used mainly for transcript annotation and resource construction [26,43,68,69]
Which RNA modifications change?	MeRIP-seq, higher-resolution assays or direct RNA sequencing	Modification-enriched regions, candidate sites or model-inferred native-RNA signals	Emerging: concentrated mainly on m^6^A [42,45,47,48,49,70,71,72,73]
Which cell population carries a signal?	scRNA-seq or snRNA-seq	Cell types, states and cell-specific expression	Expanding: strongest in immune and muscle tissues [57,59,74]
Where is the signal located?	Spatial transcriptomics	Spatially resolved expression and tissue-region context	Very limited: poultry applications have been reported in the developing chicken heart and breast muscle [54,56,60]; feasibility has also been demonstrated in non-poultry birds [75]
What is the secondary structure of an RNA, and which regions are accessible?	Transcriptome-wide structure probing (DMS-seq, icSHAPE)	Nucleotide accessibility and reactivity-derived structure models	Frontier: probing methods established outside birds [20,67]; no poultry application identified; term set shared with interaction and translation in Table 1
Which proteins bind an RNA?	CLIP or eCLIP	RNA–protein binding sites	Very limited: one chicken application (CELF1, embryonic heart) [61]; no transcriptome-wide avian binding map
Is an RNA ribosome-engaged, and what is its translational efficiency?	Ribosome profiling	Ribosome occupancy and translational output	Frontier: 13 records for the poultry term set (Table 1); sparse avian evidence

### 3.1. Bulk Transcriptome and Regulatory RNA Analysis

Short-read RNA sequencing enabled transcriptome-wide expression analysis and remains the most extensively used technology in poultry RNA-omics research. Following alignment and quantification, the sequenced reads are primarily used to estimate the relative abundance of genes or transcripts [2]. By modifying library preparation strategies, the analytical target can be expanded to various classes of non-coding RNAs. For example, small RNA sequencing is tailored for detecting mature miRNAs, while total RNA sequencing or circRNA-enrichment protocols are used to identify circRNAs. Different libraries capture entirely different categories of RNA [76,77]. Poly(A) enrichment predominantly captures mature mRNAs and polyadenylated lncRNAs but will inevitably miss most miRNAs and many circRNAs; size-selected small RNA libraries detect mature miRNAs but fail to capture their primary transcripts. circRNAs are typically analyzed from rRNA-depleted or non-poly(A)-enriched RNA [78], or through specific enrichment of circular molecules using exonucleases like RNase R, followed by identification via reads supporting back-splice junctions [79]. Consequently, the library type and enrichment method dictate which RNA classes can be detected before sequencing, and they strongly shape detection sensitivity and quantitative bias. The level of quantification also affects how results are interpreted. Gene-level analysis aggregates reads or transcript estimates originating from the same genomic locus and is generally more stable [80,81]. Transcript-level analysis requires assigning reads to distinct isoforms, so it depends heavily on annotation completeness, read length, and sequence divergence between isoforms. These two levels of results reflect total gene expression and isoform usage, respectively, and cannot be interpreted interchangeably. Building on abundance estimation, further analyses such as differential expression, co-expression modules, and competing endogenous RNA (ceRNA) networks can be conducted. The ceRNA model, initially proposed in mammalian studies [82], is typically constructed based on sequence relationships and expression correlations among lncRNAs, miRNAs, circRNAs, and mRNAs. Compared to single-gene studies, these methods allow poultry researchers to examine synergistic RNA changes on a genome-wide scale, although their foundational data remain primarily tissue-averaged abundances.

Applications of these technologies in poultry are abundant, particularly in studies regarding production traits. circRNAs have attracted attention as regulatory RNAs in muscle research [79]. For instance, circIGF2BP3 promotes the proliferation and differentiation of chicken primary myoblasts [83]; integrated analyses of circRNAs, miRNAs, and mRNAs have been employed to construct myogenesis-related ceRNA networks [36]; and lncRNA and circRNA expression profiles have been used to distinguish oxidative from glycolytic myofibers [37,38]. Research on lncRNAs further extends to lipid metabolism regulation and antiviral responses during viral infections [84], while miRNAs feature prominently in regulatory networks constructed in poultry production and health studies [41]. In contrast, the annotation and functional study of enhancer RNAs (eRNAs) remain sparse. While genome-wide predictions are now available in chickens [85] and some candidate eRNAs have been linked to potential target genes via CRISPR-mediated activation [86], existing reviews have summarized the roles of ncRNAs in poultry skeletal muscle and other production traits [87,88]. However, because most enhancer RNAs are of low abundance, highly unstable, and lack poly(A) tails, they are difficult to capture effectively with standard poly(A)-enriched or rRNA-depleted RNA-seq. Reliable identification usually requires specialized methods such as nascent RNA sequencing (e.g., GRO-seq, PRO-seq) or CAGE-seq [89,90]. Consequently, existing eRNA evidence in poultry still largely relies on computational predictions based on genomic features rather than direct measurement of nascent transcription.

Four boundaries must be considered when interpreting these results. First, standard differential expression analyses (e.g., DESeq2 or edgeR) [91] only demonstrate that RNA abundance changes under specific experimental conditions, and co-expression analyses such as WGCNA [92] reflect synchronized changes between genes. Neither can independently prove a regulatory relationship or a direct physical interaction. Second, ceRNA and “miRNA sponge” relationships are typically proposed based on binding site predictions and expression correlations, not direct measurements of molecular binding. Quantitative studies in mammals have shown that ceRNA effects are constrained by stoichiometric conditions, including the abundance of miRNAs and target RNAs, as well as binding affinities [93,94]. Therefore, in the absence of binding assays or functional perturbation, many reported sponge relationships should still be viewed as candidate mechanisms [82,95]. Third, expression differences in bulk tissue can result from changes in cell-type proportions rather than necessarily indicating transcriptional regulation within a specific cell type. Computational deconvolution can partially correct for this but cannot entirely eliminate the related uncertainties [7]. Fourth, the annotation of lncRNAs, circRNAs, and eRNAs remains unstable. Non-coding loci lack standardized nomenclature, reliable mapping across different genomes and annotation versions is often missing, and circRNA detection is highly sensitive to the choice of analytical tools [96]. This makes it difficult to align novel transcripts identified across different studies and limits cross-study comparisons. Overall, while bulk transcriptome and regulatory RNA analyses generate rich abundance data and candidate regulatory networks, the relationships that have undergone rigorous molecular and functional validation remain limited.

### 3.2. Long-Read and Direct RNA Sequencing

Total gene abundance cannot elucidate which specific transcripts a gene produces. Alternative splicing, alternative transcription start sites, and alternative polyadenylation (APA) can generate isoforms with divergent coding capacities or regulatory features. These phenomena were first mapped systematically on a transcriptome-wide scale in mammals [8]. While short-read RNA sequencing can quantify gene and exon-level abundance, it generally struggles to reliably reconstruct full-length isoforms. Consequently, before single-molecule long-read sequencing platforms became available [97], poultry transcript structures could rarely be resolved on a large scale [98]. The primary shift brought by long-read sequencing is moving the analytical focus from total gene expression to the specific transcripts and isoform compositions generated by individual genes.

Different long-read platforms prioritize different types of information and performance metrics. PacBio Iso-Seq yields highly accurate consensus reads and suits the resolution of transcript structures. ONT cDNA sequencing can often generate long reads at higher depth. ONT direct RNA sequencing reads native RNA molecules directly through nanopores, without a cDNA intermediate [46]; this preserves molecular signals that may be lost during reverse transcription. Platform selection involves balancing throughput and cost, single-base accuracy, quantitative reliability, and native RNA information. ONT cDNA is typically more advantageous regarding throughput and cost, whereas Iso-Seq excels in single-base accuracy. The quantitative performance of different long-read library strategies is influenced by sequencing depth, molecular integrity, and library preparation biases. Direct RNA sequencing is often constrained by lower throughput and insufficient coverage of low-abundance transcripts, while cDNA methods can introduce biases related to reverse transcription and PCR. Because no single platform meets all requirements simultaneously, long-read data are frequently combined with short-read RNA-seq.

Long reads also do not guarantee full coverage of every RNA molecule. RNA degradation, incomplete reverse transcription, and coverage biases can all lead to truncated transcripts; thus, the term “full-length transcriptome” remains an approximate description [99], and the final isoform catalogs heavily depend on the chosen analytical tools. In joint analyses, long reads are primarily used to define the structural architecture of transcripts originating from a locus, while higher-depth short reads are employed for abundance estimation and splice junction support. This assigns structural resolution and quantification to the data types best suited for each. ONT sequencing of 19 chicken tissues identified tens of thousands of isoforms, many absent from existing annotations, and achieved higher isoform classification accuracy than short-read methods [26]. Related benchmark studies have further compared the abilities of different tools to recover and quantify isoforms [13,44], and early Iso-Seq studies of embryonic chicken hearts confirmed that analytical pipelines substantially alter transcript identification outcomes [43]. Long-read sequencing is now expanding into waterfowl research. Multi-tissue PacBio Iso-Seq data in ducks established a full-length reference transcriptome, revealing thousands of alternative splicing events and lncRNAs missing from current annotations [68]. ONT sequencing of duck embryonic myoblasts has characterized alternative splicing dynamics during muscle differentiation [69]. These studies demonstrate that poultry, including chickens and ducks, are beginning to acquire the foundational resources necessary for transcriptome research at the isoform resolution.

Isoform-level resolution can fundamentally alter how trait-associated expression changes are interpreted. A gene showing “no change” at the overall gene level may still undergo an isoform switch that affects coding capacity or regulatory features, a change detectable only at transcript resolution [8]. However, discovering an unannotated isoform is not synonymous with proving its involvement in phenotypic regulation [32]. In the long-read poultry studies included in this review, the primary outcomes are largely transcript discovery and annotation, with direct functional validation of phenotype-associated isoforms remaining scarce [26,32,68]. Direct RNA sequencing can also preserve native molecular signals related to RNA modifications, but modification calling relies on ionic current signals and computational models, and the results still require validation by independent methods [15,45,46,99]; this will be further discussed in Section 3.3. Isoform validation is feasible in avian systems, but each available route carries a specific weakness. Isoform-specific RNA interference or antisense oligonucleotides can target a unique exon junction, but chicken primary myoblasts, hepatocytes and granulosa cells tolerate repeated transfection poorly, and shared-portion knockdown is hard to exclude. RNA-targeting CRISPR-Cas13 can in principle discriminate isoforms, yet junction-targeting guides also act on the linear parent transcript, so a Cas13 phenotype does not establish which isoform was responsible [100,101]. Ectopic expression of one isoform avoids the specificity problem but alters stoichiometry and, in birds, is performed in cultured cells, so the readout is cellular, not a production trait. The chicken *PLIN1* locus carries five verified transcript variants encoding four amino-terminally divergent proteins; nine further annotated transcripts remain computational predictions [102]. Chicken *PPARγ* produces five 3′ untranslated region isoforms by alternative polyadenylation that differ in translational efficiency without altering transactivation [103]. Its two protein isoforms differ in their effects on preadipocytes [104]. Dominant-isoform analysis separates fast- from slow-growing breeds in breast muscle [105]. The prolactin receptor shows most directly why an isoform can matter independently of transcript abundance: the sex-linked late-feathering allele carries a partially duplicated copy encoding a carboxy-terminally truncated receptor that attenuates signalling in the embryo, together with a 5′ untranslated region splice variant that raises translational efficiency after hatching without altering the coding sequence, so the two variants act in opposite directions at different stages and neither effect is visible in gene-level expression [106]. The same applies upstream in the same endocrine axis, where the goose dopamine D2 receptor, a broodiness candidate acting through prolactin secretion, is transcribed as four alternatively spliced variants whose tissue distributions differ, so a gene-level measurement in pituitary, ovary or hypothalamus averages over transcripts that are not present in the same places [107]. An isoform switch is therefore not always a quantitative variation on one mechanism: a variant that loses an amino-terminal segment or a signal peptide can change where the product acts rather than how much of it is present, so testing it requires a readout matched to the proposed mechanism rather than total gene expression.

The field remains constrained by incomplete reference transcriptomes, differences in library preparation and platform, and dependence on analytical pipelines [13,44]. Recent pore chemistry and base-calling models have improved read accuracy. Context-dependent insertion and deletion errors nevertheless persist in direct RNA sequencing and affect open reading frame prediction, allele-specific analysis and modification calling [99]. Isoform calls are limited by molecular integrity, transcript end definition, depth and algorithm choice; abundance estimates are further affected by library bias and by read assignment among similar isoforms. Most trait-associated isoforms should therefore be treated as candidate transcripts awaiting independent structural verification, functional perturbation and phenotypic validation.

### 3.3. Epitranscriptomic Technologies

RNA chemical modifications constitute an entirely different layer of information from sequence and abundance. Currently, poultry epitranscriptomic research predominantly focuses on N^6^-methyladenosine (m^6^A). This modification is catalyzed by the METTL3-METTL14 complex and recognized by specific reader proteins. While m^6^A does not alter RNA sequences, it influences splicing, stability, localization, and translation, with precise effects depending on the cell type, the specific transcript, and the reader protein involved [108,109]. Importantly, different epitranscriptomic methods measure different targets, and their results should not be treated as equivalent tiers of modification evidence.

MeRIP-seq, which uses antibodies to enrich m^6^A-containing RNA fragments, was one of the earliest methods employed to map transcriptome-wide m^6^A landscapes [16]. Its results generally manifest as modification enrichment peaks rather than precise single-base sites, with each peak potentially spanning dozens to hundreds of nucleotides. When multiple isoforms share the same region, peak signals often cannot be assigned to a specific transcript. The quantitative capacity of this method is also constrained; antibody batches, sample quality, sequencing depth, and peak-calling parameters can all impact final results, leading to only partial overlap between peaks identified in different experiments or studies [34]. Moreover, MeRIP-seq must be interpreted in conjunction with an input RNA-seq library because immunoprecipitation signals are jointly influenced by modification levels and transcript abundance. An increased peak signal in one condition could reflect an actual change in methylation level, or it might simply reflect an upregulation in the corresponding RNA’s expression. Therefore, comparisons between conditions must focus on enrichment changes relative to input expression, rather than relying solely on raw peak intensity. High-resolution or single-base methods can further narrow the range of candidate sites, and targeted experiments can validate specific loci, as demonstrated with the conserved m^6^A site in the chicken β-actin zipcode [50]. Native RNA nanopore sequencing can also infer RNA modifications using ionic current shifts or base-calling deviations. Studies have reported candidate m^6^A and m^5^C signals in chicken ceca following *Campylobacter jejuni* infection using this approach [45]. However, these results rely on trained models and algorithms rather than direct chemical assays of the modifications; different tools can also yield highly discordant sets of candidate sites [51]. Thus, antibody-enriched peaks, high-resolution candidate sites, independently validated sites, and nanopore-model inferred signals should be interpreted separately as fundamentally different forms of evidence.

To date, poultry m^6^A research has explored the egg-laying process [47], embryonic gonadal development [70], preovulatory follicle selection [48], fat deposition differences in broilers [110], the coordinated changes in m^6^A and miRNAs in embryonic breast muscle [42], and m^6^A remodeling in ALV-J-induced tumorous livers [49]. These studies frequently measure the expression of modifying enzymes like METTL3 and FTO. However, a change in enzyme levels does not directly prove that the modification status of a specific transcript has changed. Enzyme abundance, target selection, and downstream effects mediated by reader proteins represent distinct mechanistic steps that must be independently verified. The clearest functional evidence thus far comes from muscle research. For example, METTL3 promotes the translation of circSIK2 and enhances chicken myoblast proliferation [71]. METTL3-dependent m^6^A modification of *GHR* mRNA also regulates mitochondrial biogenesis during myoblast differentiation [72]. These studies rely on more than just peak landscapes; they include subsequent validation of modifying enzymes, specific transcripts, or related phenotypes. Peak-level MeRIP-seq alone only indicates regional enrichment. It does not yield the modification stoichiometry of individual sites, nor is it sufficient to establish functional causality [16]. Because RNA is fragmented during library preparation, peaks within shared sequence regions generally cannot be resolved for isoform-specific interpretation [34]. If distinguishing heterozygous loci exist in the sample and both input and IP libraries have sufficient coverage, allele-specific enrichment can be analyzed, though such results require specialized statistical handling and experimental validation [111,112].

This field currently faces two primary constraints. First, the resolution of peak-level MeRIP-seq is insufficient to reliably differentiate isoform-specific or allele-specific modifications. Second, how different m^6^A reader proteins dictate the fate of specific transcripts in poultry is mostly inferred from mammalian studies; direct evidence of RNA–protein binding in avian species remains lacking (a point elaborated in Section 3.5). Beyond m^6^A, modifications such as m^5^C, pseudouridine, and RNA editing are rarely studied in poultry and are better viewed as future research frontiers rather than systematically resolved fields. Notably, single-base resolution absolute quantification methods like GLORI and m^6^A-SAC-seq have been developed in mammalian studies, providing site-by-site m^6^A methylation stoichiometry across the transcriptome. Adopting these technologies represents a critical next step for poultry epitranscriptomics to break through the resolution limits of peak-level assays and move toward single-base absolute quantification [113,114].

### 3.4. Single-Cell and Spatial Transcriptomics

Most of the aforementioned technologies measure bulk signals averaged across diverse cells within a tissue. With the scaled application of droplet-based cellular barcoding, single-cell RNA sequencing (scRNA-seq) can record expression profiles for individual cells; spatial transcriptomics retains the coordinate information of spatial spots, tissue regions, or single cells, depending on the platform [17,115]. These methods empower researchers to trace tissue-averaged expression back to specific cell types, cellular states, and their spatial distributions, an approach now in use in poultry research [54,60]. Their direct measurement readouts are typically the gene expression of single cells, nuclei, or spatial spots, whereas cell types, developmental trajectories, and cell–cell communication are inferred computationally based on these readouts.

scRNA-seq and single-nucleus RNA sequencing (snRNA-seq) analyze different pools of RNA. Droplet-based scRNA-seq captures cytoplasmic RNA well, but tissue dissociation can result in the loss of large, fragile, or hard-to-dissociate cells. snRNA-seq analyzes nuclear RNA, making it more suitable for frozen tissues and samples like muscle and fat, where intact single-cell suspensions are particularly difficult to obtain. Compared to scRNA-seq, snRNA-seq generally yields a higher proportion of intronic and lncRNA reads and may preserve cell types typically lost during dissociation [64]. It is also essential to distinguish between directly measured expression and subsequent computational inferences. Pseudotime analysis orders cells based on transcriptional similarities to map potential state transitions, but this ordering is not a direct recording of true developmental time, and different algorithms can yield different trajectories from the same data [116]. Similarly, cell–cell communication analyses based on ligand–receptor databases only propose potential signaling interactions; they cannot prove that molecular communication actually occurred, and the results are heavily influenced by the choice of algorithm and database [117]. These technologies also have inherent measurement limitations. Standard droplet-based scRNA-seq typically has a 3′- or 5′-end bias, restricting analysis mostly to the gene level, thus failing to independently resolve full-length isoforms or RNA modifications in single cells. Spot-based spatial transcriptomic platforms often capture multiple cells within a single spot, necessitating computational deconvolution to estimate cell composition; the accuracy of this relies heavily on algorithms and reference data [7]. Imaging-based spatial methods can achieve near-single-cell resolution but typically interrogate only a predefined, smaller panel of genes.

Cell type annotation presents another challenge in poultry research. Current analyses frequently borrow mammalian marker genes, yet some poultry cells lack species-validated markers, and orthologous genes may not exhibit identical expression patterns. Consequently, cell clusters often have to be named based on morphology, expression features, and cross-species analogies, resulting in varying degrees of confidence in cell type annotations. Poultry single-cell research also faces a unique barrier absent in mammals: mature avian red blood cells are nucleated (nRBCs) and transcriptionally active. In droplet-based scRNA-seq of blood or vascularized tissues, they produce substantial amounts of hemoglobin-related reads that consume the majority of sequencing throughput and interfere with cell clustering. The mitigation strategies in common use are not equivalent, because each changes which cells survive. Fluorescence-activated sorting removes erythrocytes most completely and enriches a defined leukocyte population. It costs viability, induces stress-response transcripts and discards cell types outside the sorting panel, so atlases built this way cannot report on the cells excluded upstream [118,119]. Avian-specific erythrocyte lysis is faster and preserves the unsorted composition. Nucleated avian erythrocytes lyse less predictably than mammalian ones, so residual haemoglobin reads and lysis-derived ambient RNA are common and fragile rare populations may be lost. Single-nucleus sequencing bypasses intact erythrocytes and works on frozen tissue, so it suits fibrous and lipid-rich tissues and embryonic liver [59,120], but reads nuclear RNA only and loses cytoplasmic transcripts. Blood and vascularized tissues therefore favour sorting or lysis with the residual erythrocyte read fraction reported; muscle, adipose and archived tissues favour single-nucleus protocols. Spatial transcriptomic platforms fall into two main categories, each with trade-offs. Sequencing-based spatial barcoding methods (e.g., Visium, Stereo-seq [121]) provide unbiased whole-transcriptome information, but traditional spatial spots encompass multiple cells. Their latest iterations (e.g., Visium HD) have reduced capture areas to the micron scale, approaching subcellular or single-cell resolution [66]. Imaging-based in situ hybridization methods (e.g., Xenium, MERFISH [65]) offer near-single-cell or subcellular resolution but are generally constrained to analyzing pre-designed, limited gene panels dependent on probe library capacity. Current inferences about cell–cell communication in poultry depend heavily on ligand–receptor databases built from human and mouse data (e.g., CellPhoneDB [122]). However, the repertoires of immune ligands and receptors, such as avian chemokines and their receptors, diverge substantially from those of mammals and lack complete correspondence in gene number and family composition [123]. Directly applying mammalian databases is therefore prone to yielding false-positive or false-negative communication inferences. No curated avian ligand–receptor database is available, and renaming orthologues does not solve the problem, because differences in gene number and family composition leave some mammalian pairs without an avian counterpart and some avian receptors without an entry to inherit. A workable interim strategy is a restricted, species-anchored list built on the annotated chicken chemokine and receptor repertoire [123]. Mammalian pairs are retained only when both partners have a one-to-one avian orthologue with conserved domain architecture. Surviving predictions are hypotheses, tested by showing expression of both partners in the proposed sender and receiver cells. Studies using an unmodified mammalian database should report its version and the proportion of pairs mappable to avian orthologues [117,122].

Despite these limitations, single-cell and spatial methods have substantially improved the cellular resolution of poultry tissues. Developmental and organ atlases now cover the heart [54], limb bud [55], and retina [53], extending into behavioral and neural tissues [124], and a comprehensive framework for poultry single-cell atlases is emerging [28]. Single-nucleus sequencing has further identified myoblast subpopulations associated with muscle growth [59]. Currently, the most widespread applications are in immunology, including atlases of leukocytes and PBMCs [119,125], lymphoid organs [118], and infection or challenge responses in the bursa of Fabricius [126], spleen [57], cecum [58], and PBMCs [127]. Comparisons between disease-resistant and susceptible individuals have linked variations in cell composition and states to resistance phenotypes [74,128]. Spatial transcriptomics remains far less used: the poultry term set returns 15 records (Table 1), and poultry applications currently include the developing chicken heart and breast muscle [54,56,60]. Spatial and single-nucleus profiling of the avian optic tectum in non-poultry birds shows the approach transfers [75], so the limit is study design and platform access, not species feasibility. These results can indicate what cells or tissue regions a signal originates from, but spatial localization alone does not prove the functional role of the signal. Overall, while single-cell and spatial transcriptomics contribute essential cellular origin and spatial location data to poultry research, their interpretations are jointly dictated by sample processing, computational inference, cell annotation accuracy, and platform resolution.

### 3.5. RNA Structure, RNA–Protein Interactions, and Translation

RNA structure, RNA–protein interactions, and translation states further broaden the scope of RNA-omics, but their application in poultry remains highly limited. Transcriptome-wide chemical probing methods, such as DMS-seq and icSHAPE, investigate RNA structures and their dynamics by measuring nucleotide accessibility [20,67]. Within the search scope of this review, no reports of these methods being applied transcriptome-wide to poultry hosts were found. The study of m^6^A-dependent ZBP1 binding in the chicken β-actin zipcode [50] provides evidence of RNA–protein interaction at a specific locus but does not constitute transcriptome-wide RNA structure probing.

RNA-binding proteins (RBPs) are integral to splicing, stability, localization, and translation regulation, yet poultry research still lacks systematic, transcriptome-wide RNA–protein interaction maps. Taking the m^6^A reader mechanisms discussed in Section 3.3 as an example, studies using cross-linking immunoprecipitation methods like CLIP or eCLIP to directly map the binding sites of poultry reader proteins are exceptionally rare [62], despite these methods being widely employed in mammals. The execution of CLIP/eCLIP generally relies on antibodies capable of effectively immunoprecipitating the target RBP; in the absence of suitable antibodies, validated epitope-tagged RBP systems can serve as alternatives [62]. The current paucity of literature is better understood as an evidence gap in poultry research rather than a fundamental inapplicability of the technology to avian species.

Ribosome profiling (Ribo-seq) evaluates RNA translation states and efficiency by sequencing ribosome-protected fragments [19]. Compared to conventional bulk RNA-seq, its application in poultry is sparse but not entirely absent, with studies exploring chicken spleen, reproductive tissues, brain tissue, embryonic tissues, and primary myoblasts [71,129,130,131]. Its successful execution demands stringent control over the preservation of ribosome states, nuclease digestion, rRNA depletion, species-specific read mapping, and quality assessment of trinucleotide periodicity [19]. Currently, it is more accurate to view ribosome profiling as an emerging technology in poultry research rather than an unapplied one. Another emerging value of ribosome profiling is breaking the traditional boundary between “coding” and “non-coding” RNA: by detecting short open reading frames (sORFs) occupied by ribosomes, functional micropeptides can be discovered in RNAs previously deemed non-coding. One chicken study combined RNA-seq with Ribo-seq to identify a 74-amino-acid micropeptide encoded by an lncRNA, proving it regulates myoblast proliferation and differentiation [132]. This strategy could add a new functional layer to studies of poultry traits, such as muscle development.

Overall, while the methodological foundations for studying RNA structure, RNA–protein interactions, and translation states are well-established elsewhere, systematic data in poultry remain deficient. This highlights that current poultry RNA-omics evidence is heavily skewed toward expression abundance, isoforms, modifications, and cellular origins, leaving substantial room for expansion into structural, protein-binding, and translational layers. Transcriptome-wide structure probing needs microgram amounts of intact RNA per condition and a reactivity-to-structure model calibrated in the organism studied; without avian benchmark datasets, reactivity cannot be separated from degradation. CLIP and eCLIP need an antibody that immunoprecipitates the target protein under stringent conditions; antibodies against avian RNA-binding proteins are largely unavailable and rarely validated for immunoprecipitation, and expressing an epitope-tagged protein instead requires a transfectable or transgenic avian system, which returns to the germline bottleneck discussed in Section 6.4. The one published avian application, CLIP of the splicing regulator CELF1 in embryonic chicken heart, identified individual targets, not a transcriptome-wide binding map, which marks both feasibility and the ceiling [61]. Ribosome profiling needs translation arrested within seconds, species-specific rRNA depletion and an annotation complete enough to assign footprints to open reading frames. Chicken depletion reagents and short open reading frame annotation are less developed than their mammalian equivalents, so avian studies are sparse, not absent [71,129,130,131,132]. Single-base modification mapping additionally needs chemistry-specific reference standards, which exist for mammalian transcriptomes only [52,113,114]. These are reagent and resource gaps rather than biological barriers, and each has a defined remedy.

## 4. Selection and Combination of Poultry RNA-Omics Technologies

### 4.1. Choosing Technologies Based on Research Questions

**Figure 2:**
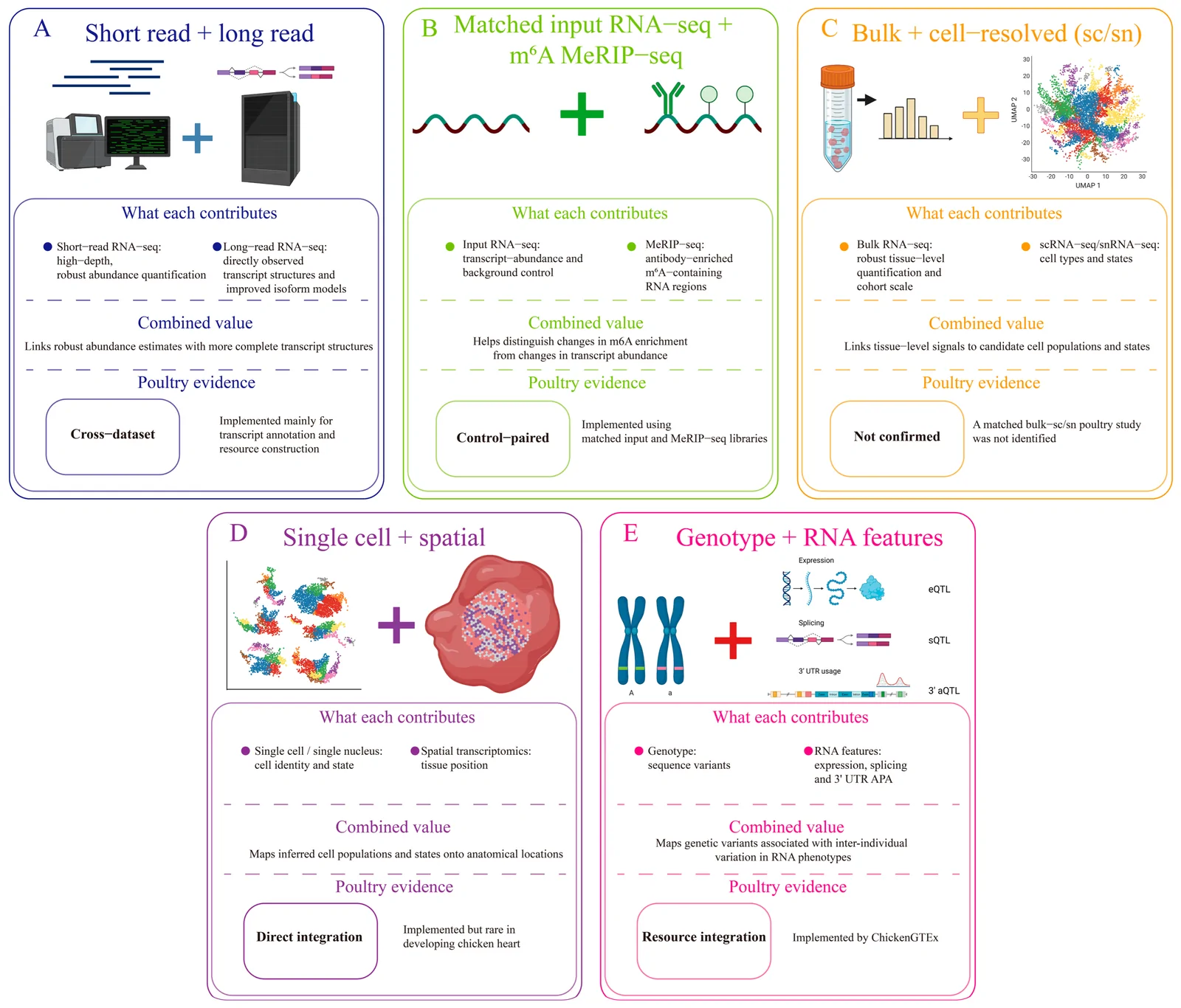
Current and prospective combinations of RNA-omics technologies in poultry. The figure summarizes complementary technology pairings, including short-read with long-read sequencing (**A**), RNA-seq with modification profiling (**B**), bulk with cell-resolved methods (**C**), single cell with spatial transcriptomics (**D**), and genotype with RNA features (**E**). The panels distinguish directly integrated or control-paired designs (**B**,**D**), cross-dataset or resource integration (**A**,**E**), and a technically feasible design for which a clearly matched poultry implementation remains to be confirmed (**C**). Every pairing addresses specific information gaps that neither individual technology can resolve alone. This figure serves as a conceptual decision-making aid rather than an exhaustive experimental workflow; refer to Table 6 for the correspondence between specific research questions and technologies, along with their implementation status in poultry.

The selection of an RNA-omics technology hinges on five interconnected factors: the specific biological question to be answered, the RNA features that must be measured, the biological material and sampling conditions available, the validation methods required to substantiate anticipated conclusions, and the cost in money, equipment and analytical capacity relative to the information returned [133]. The suitability of a technology does not depend on its novelty, but on whether its measurement readouts directly answer the research question, and whether its inherent limitations have been adequately addressed in the experimental design and data interpretation [13,133]. RNA abundance, transcript structure, chemical modifications, cellular origins, spatial locations, RNA–protein interactions, and translation states all demand dedicated measurement methods; a single platform can rarely encompass all this information simultaneously. Table 6 summarizes the correspondence between research questions and appropriate technologies, the specific RNA information each provides, and their current application status in poultry, while Figure 2 outlines potential combinations of these technologies.

Cost and infrastructure belong on this list, and they are the main reason the newer layers remain thinly represented in poultry. Per-sample charges are not reported in the primary literature and vary with country, platform and provider, so they are best treated as tiers (Table 3 and Table 5). Bulk short-read sequencing is the lowest tier, affordable at the tens-of-samples scale that trait studies require. Long-read sequencing and MeRIP-seq occupy an intermediate tier, with MeRIP-seq effectively doubled by its matched input library; droplet-based single-cell and single-nucleus platforms and spatial transcriptomics form the highest tier, commonly about an order of magnitude above a bulk library per sample once reagents, instrument access and sequencing depth are combined, and they additionally require fresh or well-preserved tissue, a platform within reach and analysts able to run the pipelines. Because much poultry research is carried out where production is concentrated but platform access is limited, the expensive layers cannot be applied at the sample sizes needed for individual-level trait inference. A pragmatic design is therefore to reserve the expensive layer for questions that specifically require it, using a small number of representative animals, while maintaining replicated contrasts across the cohort with bulk sequencing and deconvolution against the cell-resolved reference [7,134]. Per-sample costs can be reduced, where biologically appropriate, through sample multiplexing in single-cell experiments or by profiling selected sections rather than whole organs [135]. Where the required layer is not feasible, a bulk experiment accompanied by an explicit statement of the unresolved questions may provide a more transparent alternative.

### 4.2. Existing and Potential Technology Combinations in Poultry

The fundamental purpose of combining multiple technologies is to use different methods to fill complementary informational gaps, not merely to pursue a so-called “more comprehensive” research design [136,137]. Currently, the evidence base for different combinatory strategies in poultry RNA-omics is uneven [28]. Based on how the data were generated and linked, these combinations fall into three categories: directly integrated studies, in which two or more RNA layers were measured on the same or on paired samples within one study; cross-dataset comparisons, in which layers generated independently, often for resource construction, are interpreted against one another; and technically feasible but not yet implemented designs, established elsewhere but with no poultry example identified in this search. The distinction concerns sample linkage and provenance rather than how advanced the technologies are, and it determines how strong a conclusion a combination can support.

Directly integrated studies. Studies simultaneously measuring multiple RNA information layers on the same samples remain rare. Taking MeRIP-seq as an example, standard analysis inherently requires a paired input RNA-seq library to differentiate changes in modification enrichment from changes in transcript abundance [16]; this design has been deployed in poultry research [73,138]. However, because the input library serves primarily as an analytical control, MeRIP-seq and its paired input should not be strictly viewed as the integration of two independent omics layers [137]. Some studies add further measurements, such as simultaneously analyzing m^6^A, input RNA-seq, and miRNA data in embryonic chicken breast muscle [42]. As this review could not confirm whether all data types were derived from the exact same individuals or paired sample aliquots, a more cautious interpretation is that the study performed intra-study integration across corresponding sample groups, rather than a strictly paired-sample design. Linking genetic information with RNA features represents another form of combination. The ChickenGTEx project aggregated genotype, gene expression, splicing, and 3′-UTR usage data across multiple tissues, linking RNA features to genetic variants through eQTL and sQTL analyses [25,27]. This is resource integration interfaced through genotypes, rather than the direct combination of two RNA measurement platforms in a single assay. ChickenGTEx is the most complete resource of its kind in poultry, and its scope also defines its limits. Its tissue panel, breeds, ages and physiological states are those of the contributing datasets, not a designed sampling frame. Tissues and stages central to particular traits are therefore thinly represented, and discovery power is uneven across the atlas. Regulatory effects are also context-dependent, so a pooled atlas misses effects conditional on tissue, age or physiological state [139,140]. Because the underlying populations are largely research or local breeds rather than the commercial lines used in breeding programmes, a catalogued association is best treated as a prior to be tested in the target population [141]; reading the resource as a solved genotype-to-transcriptome interface overstates what it currently delivers.

Cross-dataset comparisons. Certain technological pairings in poultry are currently used predominantly for constructing reference resources or cross-referencing independently generated data. The integration of short-read and long-read data is methodologically mature: long reads define the transcript structures, while deeper short reads provide reliable quantification and splice site support [43,142]. Yet, in poultry, this pairing primarily serves transcript annotation and resource building; applications in paired-trait studies are scarce [26,43,68,69]. Benchmarking general methodologies demonstrates technical feasibility but does not substitute for actual empirical evidence in applied poultry research [13,44]. Similarly, cell-resolved data are frequently cross-referenced with pre-existing bulk tissue studies, though the two datasets may not originate from the same study or identical samples. A muscle single-nucleus transcriptome can be interpreted alongside established bulk myogenesis expression profiles [59], representing cross-dataset comprehensive analysis. Spatial datasets can also computationally infer cell compositions based on expression signatures internally [56,60]. This differs structurally from integrating an external single-cell reference, or generating paired single-cell and spatial data within the exact same study. The degree of sample correspondence directly dictates the strength of the conclusions these approaches can support.

Technically feasible but not yet implemented designs. Several designs have strong methodological foundations but have not yet become routine in poultry research. These include pairing bulk RNA-seq with scRNA-seq or snRNA-seq [134], pairing scRNA-seq with spatial transcriptomics [143], integrating long-read cDNA sequencing with direct RNA sequencing [13,46], combining single-cell with long-read isoform analysis [144], performing single-cell RNA modification profiling [145], pairing CLIP or eCLIP with expression or modification data [62], and combining ribosome profiling with isoform or modification data [19]. These strategies are well-established elsewhere, but actual implementation evidence in poultry is highly limited. Current poultry direct RNA sequencing studies can simultaneously acquire sequence and native molecular signals from a single library [45]. However, this is not synonymous with running parallel long-read cDNA and direct RNA sequencing. Only when both library types are constructed from matched samples and analyzed jointly can it be concluded that this paired design has been implemented in poultry. Ultimately, the value of multi-technology designs should be judged by the specific type of information they restore, not just the number of technologies employed.

### 4.3. Under What Conditions Does Integration Strengthen Evidence?

Whether a technological combination actually strengthens interpretation depends on whether it introduces new information relevant to the research question, rather than simply inflating the dataset size [137]. This can be evaluated across three dimensions. First is the biological and sample correspondence. Diverse measurements should center on the same biological contrast and ideally be derived from paired individuals or matching sample aliquots, rather than unrelated populations or separate studies. Because most assays are destructive, taking distinct sample aliquots from the same individual is generally more feasible than repeated assays on the exact same material; provided sampling and distribution are properly controlled, this design does not inherently compromise evidence strength [146]. Second is the complementarity of the measurements. Different technologies must provide distinct pieces of information required to solve the biological puzzle. For example, gene abundance and isoform composition, transcript expression and modification enrichment relative to input, and cell identity combined with spatial location are all inherently complementary [136]. If two assays ultimately only provide gene-level abundance changes, the result may merely be an increase in data volume rather than new RNA information [137]. Third, validation methods must align with the conclusions drawn. Validation must target the specific RNA feature or molecular relationship proposed by the integrated analysis, going beyond merely measuring the parent gene, the modifying enzyme’s expression, or broad cellular phenotypes. For instance, isoform-specific conclusions demand isoform-specific detection or perturbation [33]; RNA modifications require site- or region-level validation [34]; cell-type conclusions demand cell-resolved localization or functional assays [134]; RNA–protein interactions require direct binding assays [62]; and conclusions regarding production or disease traits must be tied to individual-level phenotypes [5].

Poultry muscle research has begun to integrate several RNA data types within single studies. Joint analysis of gene expression, m^6^A and miRNAs narrows candidate transcripts and regulatory relationships in myogenesis, and yields more concrete hypotheses than a list of differentially expressed genes [42]. Agreement between layers on the same gene or pathway does not prove the relationship, so such findings nominate candidates until specific m^6^A sites, miRNA–target interactions and their consequences are tested. Consistency also emerges across independent studies, with myogenesis separately linked to circRNA, miRNA and mRNA networks [36], m^6^A-modified myogenic transcripts [71] and fibre-type-associated ncRNA profiles [37]. Because these come from different samples, platforms and designs, they cannot be stitched into one continuous pathway. Cross-study comparison is further limited by annotation and pipelines. Incomplete non-coding RNA, isoform and modification annotation makes the same feature hard to align across datasets [26,27], and differences in reference versions, processing and experimental systems erode comparability [147]. Where genetic resources exist, eQTLs, sQTLs or modification QTLs can link RNA features to variants [25,27], and paired sampling, cellular localization and functional perturbation strengthen the link between RNA change and phenotype.

Currently, poultry RNA-omics literature features more independently generated single-layer datasets than multi-layered data derived from the same individuals or paired samples. The immediate priority should be designing complementary measurements around the exact same biological question and prioritizing the validation of specific molecular relationships proposed by multi-omics integration, rather than merely adding more technologies to the mix.

## 5. Applications of RNA-Omics in Poultry Complex Trait Research

This section asks what each technology has added to poultry complex trait research, not how regulatory mechanisms map trait by trait. The focus is how far early bulk findings have been refined by later technologies, and which domains have captured isoform, modification, cell-origin or spatial information. Studies are discussed once, in their most relevant domain, and cross-referenced elsewhere. Table 7 summarizes these applications by trait domain, technology, newly added RNA information and key limitations.

### 5.1. Production and Product Quality

Muscle growth, muscle fiber type, meat quality, fat deposition, feed efficiency, and egg quality are intensely studied production traits in poultry RNA-omics. Among these, muscle and fat research have the richest accumulation of information. Bulk transcriptomic and non-coding RNA sequencing initially expanded research from single candidate genes to regulatory networks. Developmental stage-based joint analyses of circRNAs, miRNAs, and mRNAs have been deployed to build myogenesis-related networks [36], and lncRNA and circRNA profiles help differentiate oxidative from glycolytic muscle fibers [37,38].

Fat deposition research shows the same pattern on a larger scale. Candidate ncRNA-centred networks have been proposed for hepatic lipid deposition, fatty liver, abdominal fat and the difference between intramuscular and abdominal depots [35,39,40,148,153], and joint mRNA and miRNA analyses have nominated regulators of residual feed intake in meat ducks [149]. The recurring output is a set of RNAs that co-varies with a trait or a developmental stage, assembled from expression correlation and sequence-based target prediction, so individual candidates remain nominations rather than established control points. The more informative pattern is not which gene appears in any one network but that independent studies converge on lipogenic and myogenic pathways.

Muscle development shows most clearly what the newer layers add. Epitranscriptomics reports the modification state of transcripts, as in the coordinated m^6^A and miRNA changes in embryonic chicken breast muscle [42]. Some muscle studies manipulated m^6^A writers and observed myoblast phenotypes [71,72], which is closer to functional evidence than correlation alone. These results come from cell models, so their consequences for muscle growth in the bird remain to be shown. Single-nucleus transcriptomics identifies which cell populations generate a bulk signal, and the satellite-cell subpopulation associated with myotube hypertrophy indicates that a tissue-average change can be driven by a minor subcluster [59]. Spatial transcriptomics adds position: perivascular macrophages with a distinct lipid metabolism signature localize the onset of wooden breast myopathy to a specific niche [60]. Spatial profiling has also tracked breast muscle at different growth ages [56]. Each study contributes one layer, and none has yet been assembled into a multi-layer causal chain within a single experimental system.

Evidence is unevenly distributed across production traits. Muscle growth and fat deposition draw on bulk expression, ncRNA, modification and, increasingly, cell-resolved and spatial data. Egg composition, eggshell quality, meat quality indices and feed efficiency still rest on bulk or ncRNA sequencing in a few breeds or lines [149], with isoform-level, epitranscriptomic and cell-resolved work almost absent. Multi-omics syntheses of chicken growth traits are beginning to appear [154], and where genotype resources exist, RNA features can be anchored to cis-regulatory variants or trait-associated loci [25,27]. Interpretation requires a firm separation between molecular and production phenotypes. Proliferation, differentiation or metabolic shifts in cultured myoblasts, and lipid metabolic states mapped to a cell cluster, are mechanistic evidence. Carcass yield, intramuscular fat percentage, meat quality and feed conversion are outcomes measured on birds and flocks. Most of these relationships remain correlational network inferences with perturbation limited to a few factors in culture, so converting effects validated in cells into confirmed drivers of live-animal performance remains the central gap.

### 5.2. Reproduction

Poultry reproduction depends on the development and dynamic regulation of the reproductive axis [155,156]. Its components are distinct phenomena, not one trait. Age at first egg reflects the developmental trajectory of the hypothalamus-pituitary-ovary axis, while egg production rate and persistency gauge the dynamic function of the mature axis [156]. Follicle selection depends on repeated cell-fate decisions in the ovary [155], and fertility and hatchability are further governed by oocyte quality and early embryonic development [157]. An RNA feature correlating with one of them does not automatically bear on the others, so each must be interpreted within its own tissue, developmental stage and phenotypic context.

Presently, poultry reproduction RNA-omics relies heavily on tissue-averaged expression, regulatory RNA networks, and m^6^A modifications. Whole-transcriptome sequencing has charted coordinated ncRNA shifts across the HPO axis and during ovarian atresia in broody hens [150,151]. This broadened the focus from single genes to multi-class RNA networks. However, these bulk-tissue findings fail to resolve the distinct contributions of varied cell populations. Small RNA sequencing has identified candidate miRNAs associated with reproduction, and epitranscriptomics has appended modification layers. For example, m^6^A dynamics during egg-laying correlate with divergence between high- and low-yield hens [47], and specific m^6^A-tagged genes have been implicated in preovulatory follicle selection [48].

Long-read data are rare, so isoform dynamics across the reproductive axis remain largely unmapped [43], and single-cell and spatial evidence is limited [158]. Because most studies use whole ovary, follicle or oviduct samples, expression changes linked to laying performance cannot be assigned to granulosa, theca, germ, stromal or resident immune cells [7,158]. Follicular composition also shifts markedly with maturation, so tissue-level differences between high- and low-yield hens mix compositional change with intracellular regulation. A rigorous multi-layer study of follicular development would map co-expression change, screen modification candidates, locate the cellular and spatial origin of those signals and validate the key factors functionally. Chicken follicle research provides solid expression and m^6^A evidence but needs the localization and validation steps [48].

Limitations in this domain also extend to how evidence maps to conclusions. Research demonstrating that miR-202-5p regulates steroidogenesis in goose granulosa cells provides solid cell-level functional evidence [159]. However, this does not directly represent the in vivo egg production rate, persistency, fertility, or hatchability of the individual bird. Similarly, mapping m^6^A distributions during embryonic gonadal sex differentiation yields valuable developmental insights [70] but cannot be directly deployed to explain or predict adult reproductive performance.

In summary, poultry reproduction RNA-omics has amassed a wealth of bulk expression and partial RNA modification data, yet the resolution of isoforms, cellular origins, and spatial organization is severely lacking. Future work must integrate these RNA features with strictly defined reproductive phenotypes, cellular localization, and functional assays to reliably ascertain their specific roles in reproductive trait formation.

### 5.3. Health and Resilience

When interpreting RNA-omics studies of poultry health, molecular responses induced by infection or stress must be distinguished from individual traits such as resistance, tolerance and resilience. Most of the literature describes RNA changes after infection, environmental stress or intervention. lncRNAs participate in antiviral responses [84], *Eimeria tenella* infection shifts circRNA profiles [160], and m^6^A landscapes are remodelled in ALV-J-induced tumorous livers [49]. Native RNA sequencing has flagged candidate m^6^A and m^5^C signals in chicken caeca after *Campylobacter jejuni* infection [45]. Such studies describe how the host responds, but before-and-after measurement rarely establishes whether an RNA feature determines a bird’s innate capacity to resist disease. These designs also measure the host side only. Host–pathogen research uses dual RNA sequencing to quantify host defence networks together with pathogen virulence and metabolic gene expression in the same infected sample [161]. It has been applied in chicken models to host macrophage-like cells and the parasite during *Eimeria tenella* infection [162]. In vivo dual sequencing for enteric pathogens such as *Salmonella* and *Campylobacter jejuni* remains sparse in poultry.

Single-cell transcriptomics resolves these infection responses to specific cell populations, with applications in the bursa of Fabricius [126], caeca [58] and PBMCs [127]. Its main advantage is separating shifts in cell composition from intracellular regulation. An immune activation signal in bulk tissue may arise from infiltration or expansion of leukocyte subsets, or from altered expression in tissue-resident cells; only cell-resolved data distinguish the two. Challenge, drug-treatment and immune-evasion studies capture a response under particular conditions, not a defined disease-resistance trait [163,164].

Contrasting resistant and susceptible individuals under identical challenge conditions provides a much more direct evaluation of whether RNA features dictate outcome disparities. Comparisons between Marek’s disease-resistant and susceptible birds successfully linked immune cell composition and states to the resistance phenotype [74,128]. Splenic single-cell studies have similarly unveiled breed-specific cellular responses to Salmonella infection [57]. Compared to simply profiling a single population before and after infection, this design is better suited for identifying RNA features that co-vary with disease outcomes. Nevertheless, differences in genetic background between breeds or lines can confound results, necessitating paired challenges, replication, and functional assays for definitive conclusions. What separates a resistance design from a response design is the phenotype recorded alongside the RNA measurement, not the technology. Comparisons between inbred lines that differ in documented, heritable resistance to Marek’s disease illustrate this. Tumour incidence and survival after a standardized challenge are established for the lines, so a difference in immune cell composition can be read against a resistance phenotype [74,128]. Studies of Salmonella-challenged lines that record bacterial load alongside expression allow the same distinction: load separates birds that restrict colonization from birds that mount a strong transcriptional response while remaining colonized [165]. A minimally sufficient design records, for every bird contributing RNA, the challenge dose and route, the interval to sampling, a quantitative pathogen measure, a damage or performance measure, and the line or genotype. Tolerance additionally requires damage interpreted at a given pathogen load, and resilience repeated measurement through recovery [166,167].

Pathogen load, survival, lesion severity and production loss describe different facets of host outcome and do not always move together [166,167]. A bird may restrict pathogen replication yet suffer severe immune-mediated tissue damage, or carry a high load while maintaining stable function and production. Resistance accordingly refers to limiting infection or replication, tolerance to limiting damage or performance loss at a given load, and resilience to maintaining or recovering function under perturbation. The same distinction applies to environmental stress: circRNA remodelling in thermally challenged turkey muscle satellite cells shows that these RNAs participate in temperature responses [152]. A short-term cellular response does not demonstrate a heritable heat-tolerance trait; that would require comparing genotypes or lines under identical heat load while measuring thermoregulation, tissue damage, survival or performance. Waterfowl and turkey studies broaden the range of responses covered, but their findings apply first to those species and conditions.

Poultry health RNA-omics is not short of post-infection or post-stress expression data. What it lacks are designs that can separate the health traits defined above: genetically defined resistant and susceptible populations, standardized paired challenges, precise outcome phenotyping, multiple post-infection timepoints, control for shifting cellular composition, and longitudinal observation under genotype-by-environment interaction. Because immune and stress responses are dynamic, differences in sampling time or infiltrating cell composition can change results and complicate cross-study comparison [7,168].

## 6. Common Limitations and Priority Directions in Poultry RNA-Omics

Several challenges persistently affect various RNA-omics technologies and are not unique to any single platform. Discussing these collectively helps distinguish the universal constraints of poultry RNA-omics and outlines corresponding paths for improvement. Section 3 has already addressed platform-specific issues such as MeRIP peak resolution [34], ONT single-base errors [46], and spatial spot deconvolution [143], which will not be repeated here. Instead, this section focuses on shared limitations concerning annotation resources, experimental design, analytical interpretation, and functional validation.

### 6.1. Annotation and Reference Resources

Insufficient reference resources represent a foundational bottleneck for poultry RNA-omics. Poultry transcript annotations remain incomplete [21], non-coding RNA annotations are relatively sparse [43], and isoform nomenclature and cross-version mapping lack stability [33], resulting in situations where “novel” transcripts from different datasets cannot be confidently matched [169]. This issue manifests in two primary ways. First, transcript coordinates and gene models often shift across different genome assemblies and annotation versions. When comparing across versions, researchers must perform coordinate conversions, and difficult-to-assemble regions like GC-rich zones and microchromosomes are particularly prone to mapping errors. Second, long-read and lncRNA studies frequently unearth transcripts missing from reference annotations, but without universal identifiers and repeated detection in independent datasets, it is nearly impossible to determine whether these are stable biological entities or artifacts of specific samples or pipelines [13]. Furthermore, annotations cannot be indiscriminately transferred between chickens, ducks, geese, and turkeys without empirical verification [170,171]. Because both downstream assays and data integration rely fundamentally on reference genomes and transcript annotations, the quality of these resources directly dictates the accuracy of feature identification and biological interpretation. Consequently, species-specific long-read transcript and regulatory resources [25,26,27], functional annotation projects [172,173,174], and continuously upgraded reference genomes and pangenomes [175,176] should be viewed as the critical infrastructure of poultry RNA-omics. RefSeq and Ensembl annotate the same chicken assembly differently, so gene models, transcript sets and identifiers do not correspond one to one and quantifications made against them are not interchangeable; human genomics addressed this with an explicitly reconciled transcript set [177], and no poultry equivalent exists. Annotation completeness also differs between transcript classes: re-annotating the current chicken assembly across 47 tissues substantially expanded the long non-coding RNA catalogue relative to the reference annotation [25]. A study using the reference annotation alone therefore misses regulatory RNAs that demonstrably exist. Multi-dataset analyses should report the assembly, the annotation source and its version, and re-quantify rather than merge published count matrices.

Among these resources, T2T (telomere-to-telomere) gapless genomes and avian pangenomes are especially important. Microchromosomes and GC-rich regions have historically been the most troublesome areas for transcriptome mapping and annotation. Gapless, complete assemblies and pangenomes that capture population-level variation are the foundational tools required to resolve transcriptomic mapping issues on microchromosomes [176].

### 6.2. Experimental Design and Platform Bias

Sample processing, platform-specific characteristics, and research designs collectively skew RNA-omics results, yet these factors are sometimes underreported in poultry studies. RNA integrity, preservation methods, and preprocessing dictate downstream detection fidelity. Because scRNA-seq and snRNA-seq profile different RNA compartments, with the latter capturing a higher proportion of intronic and lncRNA reads, platform differences must be factored explicitly into method selection and cross-study comparisons [64]. Tissue dissociation can induce stress-response gene expression, confounding the assessment of native biological states; for difficult-to-dissociate or fibrotic tissues like muscle, this is precisely why snRNA-seq is often the preferred choice [63].

Different platforms also introduce methodology-dependent biases. MeRIP-seq results are sensitive to antibody batches and peak-calling parameters [34]; long-read sequencing has limited quantitative power, and isoform catalogs fluctuate with filtering criteria and analytical pipelines [13,99]. Nanopore modification-calling models are typically trained on specific species and data backgrounds, requiring rigorous, specialized validation before they can be confidently applied to poultry data [51]. Research designs and statistical analyses cast an even wider net of influence. Some datasets suffer from insufficient biological replicates, and phenotypic records are often crude, substituting breed or developmental stage for measured individual traits. Technical replicates, pooled samples, or massive numbers of cells derived from a single animal are sometimes erroneously treated as independent biological replicates, thereby underestimating inter-individual variance and artificially inflating statistical significance [178,179]. For multi-layered data integration, using paired individuals, multi-tissue sampling, or longitudinal designs is generally more effective for establishing definitive links between varied RNA features. Published benchmarks give concrete guidance. In an experiment with 48 biological replicates per condition, three replicates per group recovered only 20–40% of the genes identified as differentially expressed with the full set; recovery exceeded 85% for genes changing more than fourfold, but reaching that level for all fold changes required more than 20 replicates, and at least six replicates were recommended, rising to twelve when small fold changes matter [180]. Poultry designs usually concern fold changes well below fourfold, so the common three-birds-per-group design misses many true differences and biases those it reports towards large effects. The biological replicate is the bird, so cells from one animal, technical replicates and pooled samples must be modelled as such [178,179]. Where the expensive layer cannot be replicated at that level, it should be presented as a descriptive map on a few individuals, while a replicated bulk experiment carries the statistical contrast. Effect sizes should be reported with the number of animals per group.

Avian biology also presents a species-specific challenge: unlike mammals, avian red blood cells are nucleated. In scRNA-seq, if dedicated protocols for avian erythrocyte lysis or depletion are not employed, the massive influx of background RNA from these cells will consume sequencing throughput and severely disrupt target cell clustering and annotation. Thus, during sample preparation, stringent depletion or lysis of this erythrocyte background is usually mandatory. Poultry research also harbors a unique analytical pitfall regarding sex chromosomes. Unlike mammals, which achieve global dosage compensation via X-chromosome inactivation, the avian ZZ/ZW system lacks chromosome-wide global dosage compensation, meaning Z-linked genes are generally expressed at higher levels in males (ZZ) than females (ZW) [131,181]. Consequently, in bulk RNA-seq or eQTL analyses involving mixed-sex cohorts (especially common in reproduction or mixed-sex population studies), failing to include sex as a covariate can result in a large number of Z-chromosome genes appearing as false-positive sex-related differentially expressed genes. Sex should be recorded and modelled for every sample: as a covariate in mixed-sex designs, and as a stratification factor when the question is itself sex-specific. A covariate removes the mean shift but not sex differences in variance or regulatory architecture. Z-linked and autosomal results should be reported separately in mixed-sex cohorts, because the male-to-female expression ratio is not uniform along the Z chromosome and the region around the male hypermethylated locus behaves differently from the rest [182]. Pooled mixed-sex eQTL analysis misses sex-limited regulatory effects, so mapping should be run within sex where sample size permits, and otherwise state that sex-specific regulation was not testable [131,181].

### 6.3. Computational Analysis and Result Interpretation

ceRNA and regulatory network prediction, pseudotime and cell communication analysis, nanopore modification calling, long-read isoform identification and cross-study integration all rest on computational inference. Their outputs are sensitive to tool choice, parameters and reference versions, and different methods often produce discordant candidate lists [13,44]. Reproducibility therefore depends on reporting software versions, parameters, reference genomes and annotation versions, and on access to raw data and analysis code [183]. Without these, candidate relationships cannot be replicated and annotation and platform uncertainties compound. Interpretation should accordingly separate direct readouts from computational inference, report analytical parameters and reference resources in full, and label unvalidated relationships as candidates [5]. Computational output should not carry more biological weight than its supporting data.

### 6.4. Priority Directions for Poultry RNA-Omics

The limitations detailed above crystallize into three priority directions for future development. Reference and Measurement Resources. First, complete full-length transcript annotations for poultry must be established, alongside stable, cross-version identifiers for non-coding RNAs and isoforms [26]. Concurrently, species- and tissue-specific cell atlases and a standardized chicken cell-type ontology should be constructed to eliminate the field’s heavy reliance on mammalian marker genes for single-cell annotation [141]. RNA modification research needs poultry-specific benchmarking datasets and rigorous assessments of nanopore modification-calling models [52]. For under-explored frontiers like RNA structure, RNA–protein interaction, and translation, corresponding experimental and analytical resources must be systematically built [19,62].

Integration and Reproducibility. Multi-layered RNA-omics studies should be planned from the design phase to carry out different samplings and assays around the same biological question, rather than relying on post hoc integration of separate datasets afterward [137]. Depending on the research objectives, matched sample aliquots from the same individual or sample, multi-tissue profiling, or longitudinal sampling can be used, provided robust biological replicates are maintained [141,146]. Different data layers should strictly adhere to uniform reference genomes, annotation versions, and processing pipelines. Software versions, critical parameters, and reference resources must be completely documented; whenever possible, making raw data and analysis code publicly available will aid result reproducibility and cross-study comparisons [183].

Mechanistic and Trait Validation. Downstream experiments must target the specific RNA features or molecular relationships proposed by the omics analyses, rather than falling back on simply measuring parent genes or broad surrogate phenotypes. Validation strategies should incorporate CRISPR perturbations, isoform-specific interventions, site- or region-level modification assays, and RBP-binding experiments, deployed in appropriately selected cell lines, organoids, or enteroids [86,147,184,185]. Moreover, cellular and molecular readouts must be tightly correlated with individual-level phenotypes; where appropriate populations and genotype data exist, genetic variants can be leveraged to anchor the links between RNA features and traits. By fortifying these resources and validation pipelines, poultry RNA-omics can transition from merely cataloging candidate relationships to executing concrete mechanistic testing and trait elucidation.

Translation Towards Molecular Breeding. A terminal goal of dissecting complex traits is distilling RNA-level insights into actionable tools for genetic improvement. Large-scale poultry RNA-omics resources, particularly the eQTL, sQTL, and 3′-UTR usage QTL catalogs established by ChickenGTEx, can serve as biological functional priors that flag genomic regions more likely to harbor regulatory functions [25,27]. Building on this, transcriptome-wide association studies (TWAS) can link cis-regulated gene expression directly to traits, and connect genetic variants with RNA molecular phenotypes [186]. Incorporating such functional priors or RNA molecular phenotypes into Bayesian or weighted genomic prediction models holds the potential to boost prediction accuracy beyond standard SNP panels, which is critically important for low-heritability traits like disease resistance and feed conversion ratio. The application trajectories of resources like ChickenGTEx in precision poultry breeding have been discussed [141]. However, it must be emphasized that whether such integration truly elevates breeding value ultimately depends on the scale and quality of the poultry expression resources and their independent validation within target commercial populations. None of the conditions required for this translation is yet met in poultry. The first is stability of the regulatory signal. A substantial proportion of eQTLs are tissue-restricted in the comparable cattle atlas [140], and effects conditional on age, physiological state or exposure are missed when contexts are pooled [139]. A catalogue from adult research populations therefore cannot be assumed to describe a growing broiler under commercial conditions. The second is genotype-by-environment interaction. Because the environment acts on both the RNA measurement and the trait, an effect estimated in one management system can change in magnitude or direction in another. Cattle work combining functional annotation with multi-omics for climate-resilience prediction illustrates this [187]. The third is independent validation. Gains from adding functional priors to genomic prediction are real but modest and trait-dependent, and shrink when the annotation comes from another population [188,189]. Transcriptomic information did, however, improve prediction of immunity traits in pigs [190]. A correlation between an RNA feature and a trait is therefore not a breeding tool: it carries no heritability estimate, no additive genetic variance and no established causal direction, so it cannot be expected to raise prediction accuracy, selection differential or long-term genetic gain when inserted into GBLUP, single-step GBLUP or Bayesian prediction frameworks [191]. What can enter those frameworks is a variant or molecular phenotype whose genetic component has been estimated and whose effect has been validated in the target commercial population, which is why the priority is expression resources measured in commercial lines with recorded phenotypes rather than further catalogues [141].

It should also be noted that an objective reason poultry research frequently stalls at the network inference stage is that, unlike mice, in vivo knockout models are exceptionally difficult to generate in poultry, relying heavily on the manipulation of primordial germ cells (PGCs) [192]. Precisely because of this bottleneck, employing avian organoids or enteroids for orthogonal functional validation is indispensable for advancing poultry RNA-omics [147,185]. Furthermore, the ultimate goal of RNA-omics should not be merely elucidating mechanisms, but leveraging RNA features as molecular phenotypes. By combining them with expression eQTLs and sQTLs, these features must be integrated into poultry genomic selection (GS) systems to truly become translational tools for molecular breeding [25,27]. These systems are not interchangeable; the choice depends on the question, the tissue and the resources available. Germline modification through primordial germ cells is the only route to an animal-level knockout, and therefore to a production phenotype. It is slow and restricted to laboratories maintaining cultured primordial germ cell lines and recipient flocks. CRISPR-Cas9 editing of chicken primordial germ cells carries a high genotoxic burden, while transcriptional interference in the same cells has limited efficacy. The editing step therefore remains a research problem, not a service [192,193]. Intestinal organoids and enteroids are far more accessible, can be derived from the target breed, and support host–pathogen work that is impossible in cell lines, including complete parasite life cycles [194,195]. They represent one epithelial compartment without vasculature or a full immune complement, drift with passage, and cannot report an organism-level trait. Organoids from other tissues extend the range, not the depth. Turkey oviduct epithelial organoids provide a reproductive-tract system with region-associated markers [196], but this search found no organoid system for skeletal muscle, adipose tissue or the follicular hierarchy. These are precisely the tissues underlying the traits most often studied by RNA-omics. A realistic ladder is therefore to establish the relationship in a primary or immortalized avian cell system, reproduce it in an organoid of the relevant tissue where one exists, and reserve germline modification for the relationships that must be tested in the animal.

## 7. Conclusions

Poultry RNA-omics has evolved from profiling tissue-averaged abundance and regulatory RNA landscapes to resolving transcript isoforms, RNA modifications, cellular origins, and spatial distributions. Because different technologies prioritize distinct RNA features and are suited to fundamentally different biological questions, consistency across multiple data layers cannot, in isolation, prove causality. Therefore, accurate result interpretation demands a sharp distinction between directly measured RNA features, computationally inferred candidate relationships, and experimentally validated functional effects. Future research must select and combine technologies tailored to specific biological questions, continue refining annotation resources and analytical pipelines, and employ validation methodologies commensurate with their conclusions, thereby building reliable causal links between complex RNA features and precisely defined poultry traits. For the breeding application, this means treating RNA features as molecular phenotypes whose genetic component has been estimated and validated in the target population, rather than as correlations awaiting reinterpretation.

## Figures and Tables

**Table 1:** Search blocks, records identified in PubMed and records cited in this review. Each block combines the poultry term set (chicken OR *Gallus gallus* OR duck OR goose OR turkey OR quail OR broiler OR hen OR avian OR poultry, title/abstract) with the layer terms listed, restricted to records published up to 1 July 2026. Counts were retrieved on 22 August 2026 and are reported as descriptive search yields rather than fixed bibliometric totals. Web of Science and Google Scholar were searched with the same term sets but are reported as strategies, because Google Scholar returns no stable record totals. Counts are given so that readers can judge the coverage of each layer and the basis of the comparative statements made here.

RNA Information Layer (Search Block)	Layer Terms Combined with the Poultry Term Set	Records Identified (Total; Since 1 July 2021)	Records Cited in This Review
Regulatory and non-coding RNA	non-coding RNA OR lncRNA OR circRNA OR miRNA OR enhancer RNA OR ceRNA	1411; 609	27
Alternative splicing and transcript isoforms	alternative splicing OR transcript isoforms OR splice variants OR alternative polyadenylation	671; 80	11
Long-read and direct RNA sequencing	long-read OR Iso-Seq OR nanopore OR PacBio OR direct RNA sequencing OR full-length transcriptome	430; 326	5
RNA modifications and epitranscriptomics	m^6^A OR N^6^-methyladenosine OR epitranscriptome OR MeRIP OR RNA modification OR m^5^C OR pseudouridine OR RNA editing	154; 84	11
Single-cell, single-nucleus and spatial transcriptomics	single cell OR scRNA-seq OR single-nucleus OR snRNA-seq OR spatial transcriptome	626; 303	29
RNA structure, RNA–protein interaction and translation	RNA structure OR icSHAPE OR SHAPE-seq OR DMS-seq OR CLIP-seq OR eCLIP OR RNA-binding protein OR ribosome profiling OR Ribo-seq OR translatome OR RNA-protein interaction	139; 42	5

**Table 7:** Representative applications of RNA-omics in poultry complex trait research.

Trait Domain	Species/Tissue	Technology	Main RNA Information Added	Main Biological Insight	Remaining Limitation
Production (muscle)	Chicken/breast and leg muscle	Bulk ncRNA-seq	circRNA/miRNA/mRNA networks across myogenesis	Post-transcriptional network control of fibre development	Network-inferred; correlational [36,37,38]
Production (muscle)	Chicken/myoblasts	m^6^A + perturbation	m^6^A marks + writer manipulation	Functional m^6^A control of myoblast proliferation	Cellular, not organism-level [71,72]
Production (muscle)	Broiler/breast muscle	snRNA-seq and spatial	Cell-type and spatial resolution	Satellite-cell subset and macrophage lipid metabolism localised	Few tissues; descriptive [56,59,60]
Production (fat)	Chicken/liver, adipose	Bulk ncRNA + m^6^A	lncRNA/ceRNA modules; adipose m^6^A landscape	Multi-layer control of lipid deposition	Cell-line perturbation only [39,110,148]
Production (feed efficiency)	Meat duck/multiple	Bulk mRNA–miRNA	Integrated RFI regulators	Extends ncRNA regulation beyond chicken	Correlational [149]
Reproduction	Chicken/HPO axis, ovary	Whole-transcriptome	Coordinated ncRNA across axis	Multi-layer control of laying/follicle biology	Bulk only; no cell-of-origin [150,151]
Reproduction	Chicken/ovary, follicle	MeRIP-seq	Dynamic m^6^A across laying/follicle selection	Modification layer distinguishes yield	Region-level; correlational [47,48]
Health/resilience (response)	Chicken/bursa, caecum, PBMC	scRNA-seq/direct RNA	Cell-resolved and native-RNA challenge response	Regulatory reactivity to infection	Response, not resistance trait [45,58,126]
Health/resilience (trait)	Chicken/spleen, immune organs	scRNA-seq (line/breed comparison)	Cell-state composition vs. resistance phenotype	RNA feature linked to resistance via comparison	Few resistant/susceptible contrasts [57,74,128]
Health/resilience (G × E)	Turkey/muscle satellite cells	Bulk circRNA-seq	Thermal circRNA remodelling	Layers respond to temperature	Stress response, unperturbed [152]

## Data Availability

No new data were created or analyzed in this study. Data sharing is not applicable to this article.

## Abbreviations

The following abbreviations are used in this manuscript:

ALV-J	avian leukosis virus subgroup J
APA	alternative polyadenylation
GBLUP	genomic best linear unbiased prediction
CAGE-seq	cap analysis of gene expression sequencing
cDNA	complementary DNA
ceRNA	competing endogenous RNA
circRNA	circular RNA
CLIP	cross-linking immunoprecipitation
DE	differential expression
DESeq2	differential expression analysis tool based on the negative binomial distribution
Dual RNA-seq	dual RNA sequencing
eCLIP	enhanced cross-linking immunoprecipitation
eQTL	expression quantitative trait locus
eRNA	enhancer RNA
FACS	fluorescence-activated cell sorting
FTO	fat mass and obesity-associated protein
GHR	growth hormone receptor
GRO-seq	global run-on sequencing
GS	genomic selection
G×E	genotype-by-environment interaction
lncRNA	long non-coding RNA
m^5^C	5-methylcytosine
m^6^A	N^6^-methyladenosine
MeRIP-seq	methylated RNA immunoprecipitation sequencing
METTL3/METTL14	methyltransferase-like 3 and 14
miRNA	microRNA
mRNA	messenger RNA
ncRNA	non-coding RNA
nRBCs	nucleated red blood cells
ONT	Oxford Nanopore Technologies
PBMC	peripheral blood mononuclear cell
PRO-seq	precision run-on sequencing
QTL	quantitative trait locus
RBP	RNA-binding protein
Ribo-seq	ribosome profiling
RNA-seq	RNA sequencing
scRNA-seq	single-cell RNA sequencing
snRNA-seq	single-nucleus RNA sequencing
sORFs	short open reading frames
sQTL	splicing quantitative trait locus
TE	translation efficiency
TSS	transcription start site
TWAS	transcriptome-wide association study
UTR	untranslated region
WGCNA	weighted gene co-expression network analysis
